# Frequency of *de novo* variants and parental mosaicism in families with inactivating PTH/PTHrP signaling disorder type 2

**DOI:** 10.3389/fendo.2022.1055431

**Published:** 2023-01-04

**Authors:** Yerai Vado, Arrate Pereda, Africa Manero-Azua, Spanish Network for Imprinting Disorders, Guiomar Perez de Nanclares

**Affiliations:** Rare Diseases Research Group, Molecular (Epi)Genetics Laboratory, Bioaraba Health Research Institute, Araba University Hospital-Txagorritxu, Vitoria-Gasteiz, Araba, Spain

**Keywords:** iPPSD2, mosaicism, pseudohypoparathyroidism, NGS, next generation sequencing, parental origin, *de novo*

## Abstract

**Objective:**

iPPSD2 (which includes PHP1A and PPHP/POH) is a rare inherited autosomal dominant endocrine disorder caused by inactivating *GNAS* pathogenic variants. A high percentage of *de novo* cases has been suggested. In rare cases, parental mosaicism has been described, but its real frequency is unknown.

**Design:**

A retrospective study including a series of 95 genetically confirmed iPPSD2 probands.

**Methods:**

The frequency of *de novo* cases was evaluated and the distribution of the type of variants was compared according to the type of inheritance. The putative involved allele was determined by reverse transcriptase PCR (RT-PCR) or allele specific oligonucleotide RT-PCR (ASO-RT-PCR). The possibility of *GNAS* mosaicism was studied by next-generation sequencing (NGS) on the corresponding parental DNA.

**Results:**

In 41 patients the variant was of *de novo* origin and in 24 the origin could not be established. In both cases 66.67% of variants generated a truncated or absent protein whereas the rest of the variants were missense or in-frame deletion/duplication. Parental origin was studied in 45 of those patients and determined in 35. Curiously, the percentage of *de novo* variants at the paternal allele was higher than when paternally inherited (31.1% vs 6.67%). NGS detected mosaicism in three independent families: one from paternal DNA (allelic ratio 10%) and two from maternal DNA (allelic ratio 10% and 2%).

**Conclusion:**

*De novo* pathogenic variants are frequent in iPPSD2 (around 45%). Parental mosaicism is infrequent (8.11%) but should be analyzed with NGS, taking into account its importance in genetic counselling.

## 1 Introduction

Pseudohypoparathyroidism (PHP) englobes a heterogeneous group of rare (epi)genetic diseases characterized by kidney resistance to the action of parathyroid hormone (PTH), leading to the presence of hypocalcemia and hyperphosphatemia. PTH resistance is also related with some skeletal dysplasias and osseous abnormalities (1). In some cases, resistance to other hormones whose signaling pathways are mediated by the stimulating alpha subunit of the heterotrimeric G protein (Gsα) can also be detected, as is the case of the thyroid stimulating hormone (TSH), growth hormone-releasing hormone (GHRH) and gonadotropins (Gn) (2).

PHP and other related disorders share the same signaling cascade, so there are some overlapping features between them, such as the Albright hereditary osteodystrophy (AHO) phenotype, brachydactyly and/or hormone resistance (3). Because of that and the difficulty to establish a differential diagnosis, in 2016 the EuroPHP network proposed a new classification that encompasses these diseases: inactivating parathyroid hormone (PTH)/PTH-related protein (PTHrP) signaling disorder (iPPSD) followed by a numbering for specific subtypes that allows the description of both clinical and molecular features (4).

According to this new classification, nowadays PHP is now known as iPPSD2 and 3. Heterozygous inactivating mutations involving the *GNAS* exons 1–13, that encode Gsα, cause iPPSD2. When mutations occur in the maternal allele, it gives rise to what was classically named as pseudohypoparathyroidism type 1A (PHP1A, OMIM #103580) (5), associated not only with hormone resistance but also with AHO, characterized by round face, a thickset and short stature and brachydactyly. However, when the pathologic variants are found in the paternal allele, it produces what was known as pseudopseudohypoparathyroidism (PPHP, OMIM #612463) where patients do not generally develop multihormonal resistance but are reported to have AHO with short stature (6) and/or variable degrees of ectopic ossifications that may evolve into progressive osseous heteroplasia (POH OMIM #166350) (7). Recent reports show that some patients with PPHP can also develop resistance to PTH (8).

*GNAS* gene lays in a complex locus found in the long arm of chromosome 20 (20q13.2-20q13.3). This locus encodes various transcripts, generated through alternative first exons that splice to the common exon 2 of *GNAS* gene (9–11). Besides, the first alternative exons are regulated by differentially methylated regions (DMRs). The imprinting of *GNAS-NESP:*TSS-DMR is stablished in the paternal allele, so the expressed allele is the maternal one. Conversely, in *GNAS-AS1*:TSS-DMR, *GNAS-XL:*Ex1-DMR and *GNAS A/B*:TSS-DMR, the methylations occurs in the maternal allele and the paternal one is expressed (12–16). However, the first exon of the Gsα is not regulated by genomic imprinting and its expression is biallelic in most tissues, even if in some of them it is predominantly maternal (17–20) (**Supplementary Figure**).

Approximately the 70-80% of iPPSD2 cases are caused by heterozygous inactivating alterations (point variants or complete or partial gene/locus deletions) of *GNAS*. In two-thirds of these patients, the mutation occurs *de novo* or the parental origin cannot be determined (21). In the remaining 20-30% of the cases, it is not possible to identify the underlying molecular defect (22). Genetic mosaicism may be one of the causes of the pathology in that sort of patients, as in the last years, it has been described as the cause of many different genetic disorders (23).

Thus, the identification of mosaicism is crucial in establishing a disease diagnosis, assessing recurrence risk, and/or genetic counselling. Next-generation sequencing (NGS) with deep sequence coverage improves sensitivity and allows for accurate quantification of the level of mosaicism. NGS in combination with a suitable analysis pipeline enables the detection of low-level mosaicism that could be undetectable by conventional Sanger sequencing (24, 25).

The main objective of this work is to stablish de possibility of mosaicism in progenitors of patients with iPPSD2 caused by variants of *de novo* or unknown origin. This would help in genetic counselling giving information about the risk of recurrence and to get an idea of the possibility of this option for genetically undiagnosed patients.

## 2 Materials and methods

### 2.1 Sample selection

We included all iPPSD2 probands and parents (when available) referred between January 2010 and June 2022 to the Molecular (epi)Genetics Laboratory of the Araba University Hospital – Bioaraba National Research Institute (Vitoria-Gasteiz, Spain) for molecular analysis and harboring a *GNAS* pathogenic variant.

### 2.2 Nucleic acid extraction

Genomic DNA of the patient and parents was extracted from peripheral blood or buccal swabs using the QIAamp DNA Mini Kit (QIAGEN, Hilden, Germany), following the manufacturer’s instructions. RNA was obtained from peripheral blood or lymphocyte pellet of the index cases, using QIAamp RNA Blood Mini Kit (Qiagen). In some cases, PAXgene Blood RNA Kit (Qiagen) was used following manufacturer’s instructions. Clinical and molecular characteristics are described in **Supplementary Table 1**.

### 2.3 Sanger sequencing

The presence of point variants in the parents’ DNA was investigated by Sanger sequencing of *GNAS* exons and flanking intronic sequences from genomic DNA as previously described (18). Variants were considered as inherited if the mother or the father was heterozygous for the *GNAS* pathogenic variant; *de novo* origin was determined as confirmed when both parental DNA were available and were non-carriers of the *GNAS* pathogenic variant; and the variant was classified as of unknown origin if both parental samples were unavailable or the only available parental sample did not carry it. In the case of non-inherited long deletion(s) encompassing the DMRs, the parental origin was inferred from the methylation pattern.

### 2.4 Identification of parent-of-origin allele

When the variants were located at exon 2 to 13 (NM_001077488), the design took advantage of the fact that the *GNAS* locus is imprinted, and gives rise to different parent-specific transcripts, such as paternally expressed *A/B* and maternally expressed *NESP55* (**Supplementary Figure**). So, as it was described before (26), to specifically amplify the paternal allele the forward primer was designed to anneal in the A/B transcript. On the other hand, for amplifying the maternal allele, the forward primer was designed to hybridize in *NESP55* transcript (**Supplementary Table 2**). However our initial results revealed that *NESP55* expression is biallelic (at least in blood), so when the RT-PCR with the FAB_cDNA and R13_cDNA primers gives no amplification, we inferred the variant was on the maternal allele.

For variants located on the exon 1 of *GNAS*, as previous approach is not useful because it skips this exon, when the parents’ and index’s genotype of the SNP rs7121 (located at *GNAS* exon 5) was informative, an allele-specific oligonucleotide (ASO)-RT-PCR was designed (**Supplementary Table 3**). The same approach was also used (when possible) for those cases in which the FAB_cDNA and R13_cDNA approach was not conclusive.

In all the cases, RT-PCRs were performed with One-Step RT-PCR kit (Qiagen) following the manufacturer’s instructions. The thermocycling was 50° C (30 min); 95° C (15 min); 40x {94° C (1 min); 62° C (1 min); 72° C (1min)}; 72°C (10 min); 4° C (∞). In case of not obtaining enough RT-PCR product, a nested PCR was run with the most convenient combination of primers (**Supplementary Table 2**).

After the amplification, amplicon purification by ExoSAP-IT^®^ and Sanger sequencing was performed.

### 2.5 Next generation sequencing

We designed and validated a custom panel of 38 genes/regions (119,748 bp) related with differential diagnoses of PHP (PHP-like_v1). The study of this panel was conducted on the genomic DNA of the corresponding parent(s) using Nextera Flex for the enrichment method (Illumina Inc., San Diego, CA, USA) and sequenced in an Illumina’s MiniSeq platform High Output (7.5 Gb), at high coverage (mean region coverage depth of >500 x) and with a 150 bp pair-end strategy (Illumina Inc.). The MiniSeq integrated DNA Enrichment Analysis Module was used for secondary analysis (alignment with BWA 0.7.7 on GRCh37/hg19 and VCF and bam/bai files were obtained with GATK Variant Caller v1.6-23 and SAMtools v0.1.19, respectively). Downstream tertiary bioinformatic analysis of VCF files (annotation, filtering, and variant prioritization) and bam files (for CNV detections) was performed with the help of commercial software VarSeq V2.2.1 (Golden Helix, Bozeman, MT, USA). The parameters used for the filtering and prioritization were as follows: quality assessment (Q > 30; mean coverage > 50x). Bam files were also visualized with Integrative Genomics Viewer (IGV) to check for the presence of the variant already identified in the corresponding index patient.

The sample of patient PHP1125-M described before as being a mosaic carrier of a *GNAS* pathogenic variant (26), was used as positive control.

### 2.6 Statistical analysis

We compared the type of variants between the patients with an inherited pathogenic variant and the patients with a *de novo* causative variant using Chi-squared or Fisher’s exact test (depending on the sample size). A p value ≤0.05 was considered significant.

Pathogenic variants were classified into two groups: variants leading to a truncated protein (nonsense, frameshift, splice-site variants); and variants non-affecting the reading frame (missense, insertion/deletion in frame, whole gene deletions). The comparison was made first for confirmed inheritance status, and deduced involved allele.

## 3 Results

### 3.1 Estimation of *de novo* pathogenic variants frequency

In a total cohort of 95 patients with an identified variant affecting *GNAS* gene, 28 inherited the variant from their mother, 2 from their father, in 41 patients was of *de novo* origin and in the remaining 24 the origin could not be stablished due to the lack of one or both parental samples (**Supplementary Table 1**). In five cases the patients presented gross deletions affecting part of the whole *GNAS* gene (2 maternally inherited, 1 paternally inherited, 1 *de novo* and 1 of unknown origin). The parental origin of the last two deletions was inferred as being a *de novo* variant located on the maternal allele whereas the other patient carried the deletion in her paternal allele. Thus, in our series the frequency of confirmed *de novo* pathogenic variants, where both parents confirmed to be non-carriers, was estimated at 43.16% and the frequency of putative (those confirmed plus those of unknown origin) *de novo* pathogenic variants was estimated at 68.4%.

The parental origin and mutation type are shown in **Table 1**. Most of the variants (64.2%) generated a truncated or absent protein whereas a smaller percentage (36.8%) just changed or included new aminoacids without affecting the reading frame. This distribution could be observed independently of the type of inheritance, with the exception of patients who inherited the variant from their father (only two cases).

**Table 1:** Types of variants identified in the *GNAS* gene and their inheritance.

Missense		In-frame dup/del	Nonsense	Frameshift	Splicing	Gross deletion	Inversion	Total
Maternal	12 (42.9 %)	0	3 (10.7 %)	8 (28.6 %)	3 (10.7 %)	2 (7.1 %)	0	28 (29.5 %)
Paternal	0	1 (50 %)	0	0	0	1 (50 %)	0	2 (2%)
De novo	11 (26.8 %)	2 (4.9%)	6 (14.6 %)	15 (36.6 %)	5 (12.2 %)	1 (2.45 %)	1 (2.45 %)	41 (43.2 %)
Unknown	8 (33.33 %)	0	5 (20.83 %)	8 (33.33 %)	2. (8.33 %)	1 (4.18 %)	0	24 (25.3 %)
								95

The percentage of each type of variant has been calculated for the different variants according to inheritance. The percentages of the variants according to their inheritance have been calculated in comparison with the total.

### 3.2 Identification of the involved parental allele

**Figure 1:**
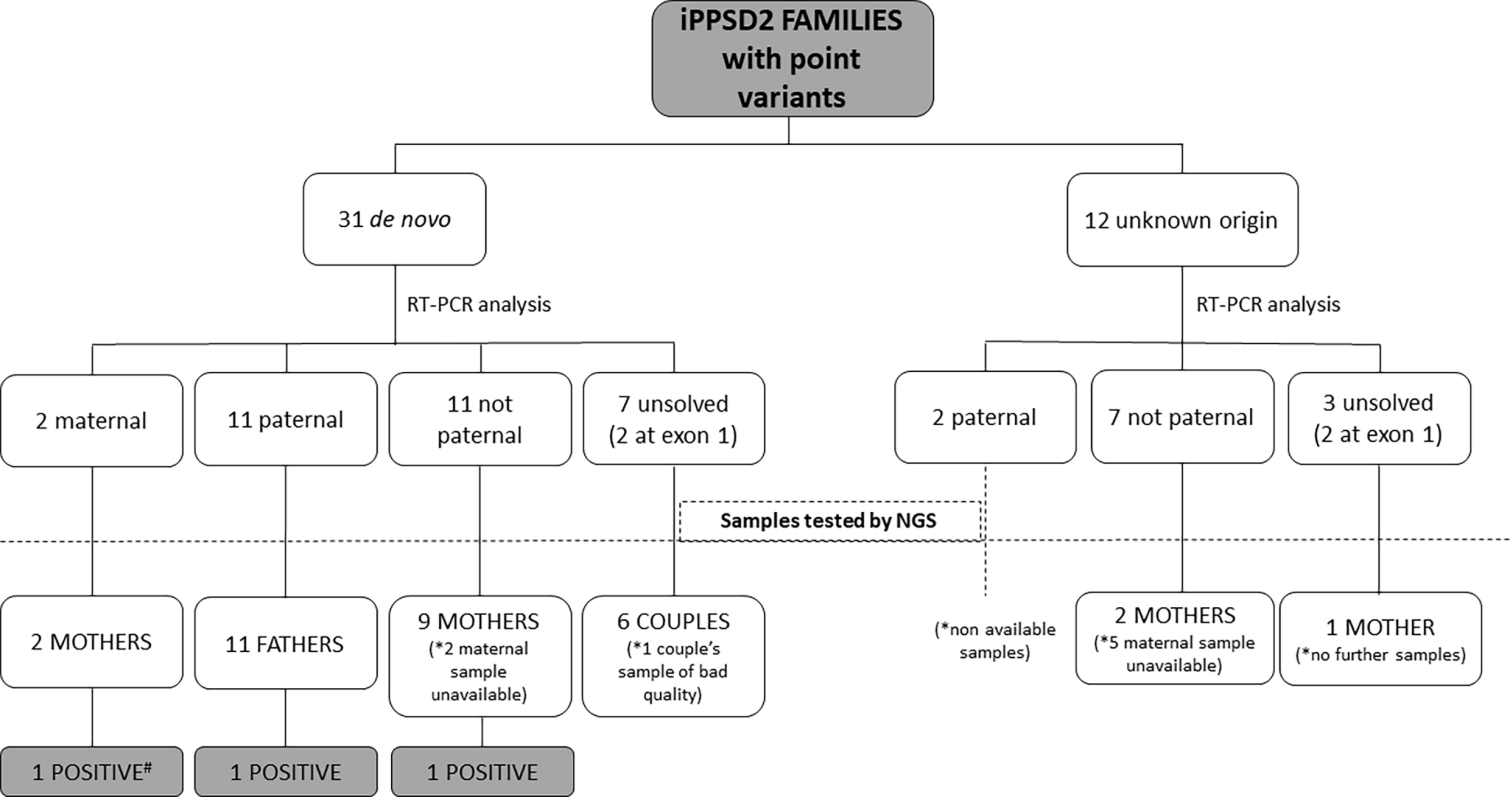
Schematic representation of the type of samples analyzed from the iPPSD2 families with variant of *de novo* or unknown origin and the obtained results. *: Number of unavailable samples for NGS studies. #: Sample used as positive control.

In 9 patients with *de novo* point variant and 11 patients with a variant of unknown origin we were not able to obtain fresh sample for the present study, so the studies were developed in the rest of the cohort (**Figure 1**).

After RT-PCR analyses of the 31 patients with a point variant of *de novo* origin, we were able to establish that in two cases it was on the maternal allele, in 11 cases on the paternal one, in 11 cases it was not detected on the paternal allele, so we inferred it was on the maternal one, and we could not resolve the involved allele in 7 of the cases (in two patients the variant was at exon 1) (**Table 2**).

**Table 2:** Description of the type of variants identified in the *GNAS* gene and the allele in which they are found in patients of variants of *de novo* or unknown origin.

Missense		In-frame dup/del		Nonsense	Frameshift	Splicing	Gross deletion	Total
De novo	Maternal allele	3 (21.4%)	0	0	9 (64.3%)	1 (7.15%)	1 (7.15 %)	14 (43.7%
	Paternal allele	6 (54.5%)	1 (9.1%)	1 (9.1%)	2 (18.2%)	1 (9.1%)	0	11 (34.4%)
	Unsolved	0	0	3 (42.9%)	2 (28.6 %)	2 (28.6%)	0	7 (21.9%)
								32
Unknown origin	Maternal allele	2 (28.6%)	0	1 (14.3%)	3 (42.9%)	1 (14.3%)	0	7 (53.8%)
	Paternal allele	2 (66.67%)	0	0	0	0	1 (33.33%)	3 (23.1%)
	Unsolved	1 (33.33%)	0	1 (33.33%)	0	1 (33.33%)	0	3 (23.1%)
								13
Total	Maternal allele	5 (23.8%)	0	1 (4.8%)	12 (57.1%)	2 (9.5%)	1 (4.8%)	21 (46.7%)
	Paternal allele	8 (57.2%)	1 (7.1%)	1 (7.1%)	2 (14.3%)	1 (7.1%)	1 (7.1%)	14 (31.1%
	Unknown	1 (10%)	0	4 (40%)	2 (20%)	3 (30%)	0	10 (22.2%)
								45

Variants located in the maternal allele were considered to be those that, after RT-PCR studies, were confirmed and also those cases in which it was ruled out that they were found in the paternal allele. In the case of gross deletions, the parental origin was determined by the methylation pattern observed after MS-MLPA study. The percentage of variant type has been calculated for the different variants depending on the allele involved. The percentages of the variants according to the involved allele have been calculated in comparison with the total of each subgroup.

Regarding the 12 cases with a point variant of unknown origin due to lack of the sample of one or both parents, two of the variants were on the paternal allele, 7 were inferred to be on the maternal allele and in 3 cases we couldn’t elucidate the involved allele (in two patients the variant was at exon 1).

Both in the case of variants located in the maternal allele and in those in which the parental origin could not be identified, most of them generated truncated protein. However, in the case of variants located on the paternal allele, slightly more than half were missense variants or variants that did not alter the reading frame.

When comparing the results of patients who inherited the variant from a parent with those who inherited it *de novo*, we observed that there was a higher percentage of patients with the *de novo* variant in their paternal allele (31.1%; or 14.5% if compared with the whole cohort) than those who inherited it from their carrier father (2%). However, we found no significant differences in the distribution of the type of variants in the parental alleles depending on whether or not they had been inherited.

Regarding clinical features, patients with the variant in the paternal allele (regardless of whether it was inherited or not) did not present resistance to PTH, while this was present in all patients with the alteration in the maternal allele, regardless of the type of inheritance (we lacked this data in one of the patients).

### 3.3 Estimation of mosaicism with NGS analysis

**Figure 2:**
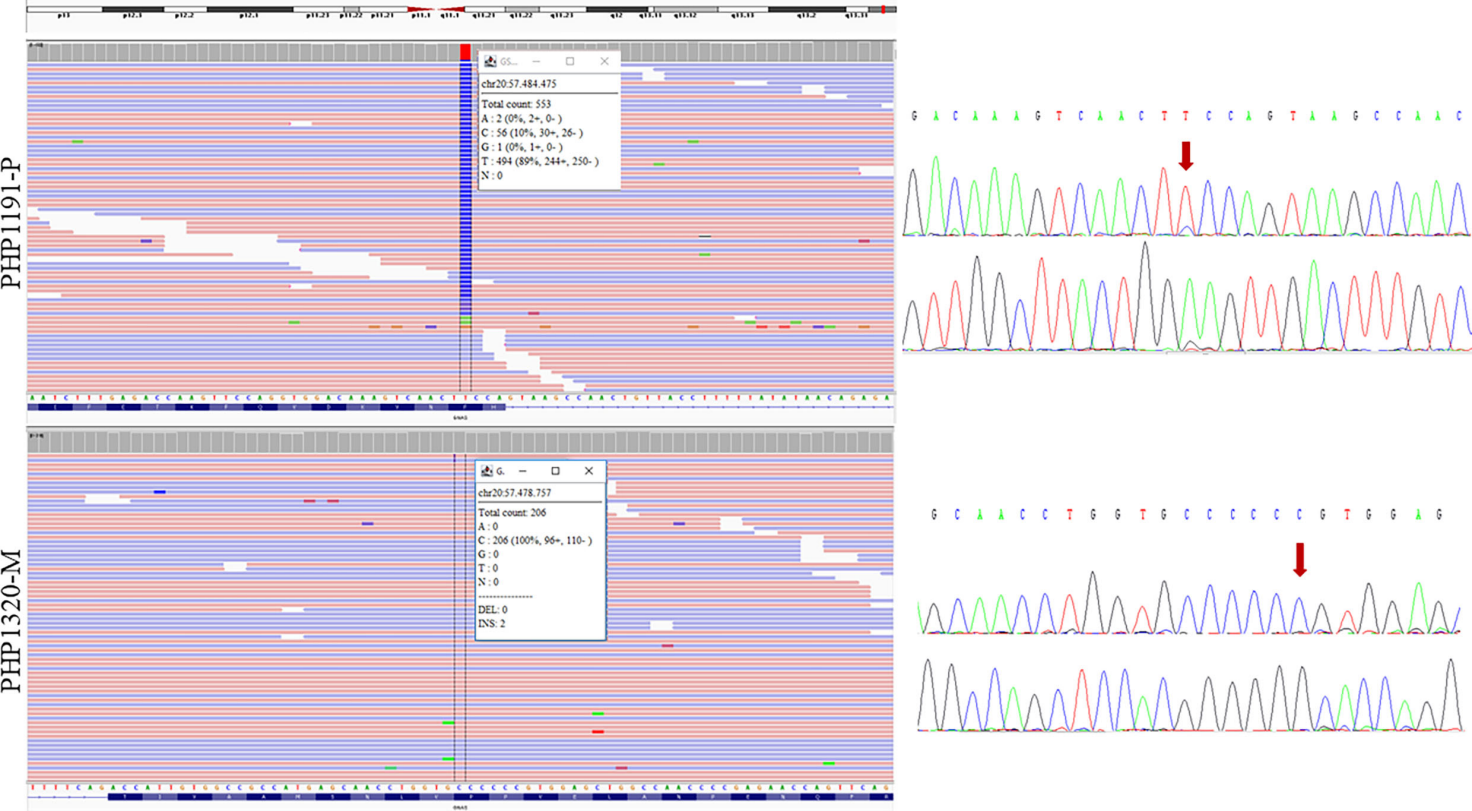
Sequencing results of the two parents carrying the mosaic variants. NGS results are shown on the left and Sanger sequencing results on the right (for each sample, upper panel for forward sequencing, bottom for reverse; the red arrow indicates the position of the change). Integrative Genomics Viewer (IGV) (27) was used for visualizing the variants identified by NGS. Reads are sort by base, showing the variant in the upper part of the reads’ track. Information in the box indicates the number of total reads at the position of interest, the count of each base at that position and the percentage of each allelic variant. PHP1191-P is an asymptomatic father whose NGS sequencing revealed the presence of the mutated C variant in around 10% of the reads; reanalysis of Sanger sequencing allowed the observation of a small peak. The duplication of a C in PHP1320-M (mother) was observed in 2 of 206 NGS reads; however it could not be observed at Sanger sequencing. The transcript used as reference was NM_001077488.2.

Once the affected allele was known, the NGS study of the corresponding progenitor’s sample was performed. That is, if the proband presented the variant in their maternal allele, the genomic DNA of the mother was studied by NGS. In the cases where the allele involved could not be resolved, samples from both parents were studied (if available) (raw data are available at the European Nucleotide Archive, submission number ERP141662). A total of 16 needed parental samples were lacking. After studying all the 37 samples of the progenitors, mosaicism was detected in two of them (besides the positive control). In the father of family PHP1191, NGS analysis revealed the presence of the pathogenic variant c.659T>C with an allele ratio of 10% in the leukocytes DNA, whereas in the mother of family PHP1320 the c.351dupC was detected in 2.4% of the leukocytes’ DNA and in less than 1% in buccal swab. The blood samples from both members were screened again by direct Sanger sequencing for the *GNAS* pathogenic variants. A small peak was observed in the chromatogram of the PHP1191-P’s leukocytes DNA but not in PHP1320-M’s DNA (**Figure 2**). Taking this into account, the percentage of mosaicism in our cohort is 8.11%.

## Discussion

Decreased expression or activity of Gsα, the α subunit of the stimulatory G protein is the most frequent cause of PHP (2). The main subtypes of PHP are caused either by *de novo* or autosomal dominantly inherited inactivating genetic mutations (known as PHP1A/PPHP or iPPSD2 according to the new classification (4)), or epigenetic, sporadic, or genetic-based alterations within or upstream of *GNAS* (PHP1B/iPPSD3) (4, 21). iPPSD2 patients carrying either point or structural pathogenic variants on the maternal allele show AHO with resistance to multiple hormones (i.e, PHP1A), whereas when present in the paternal allele, the same mutations lead to AHO usually in the absence of endocrine abnormalities (PPHP) or may lead to heterotopic ossifications progressively extending into skeletal muscle and deep connective tissues (POH) (2). So, as the clinical implications may be different depending on the parental origin of the variant, and some of them as PTH resistance and brachydactyly evolves with time, parental testing is indicated when a genetic alteration is detected. Mutations can be either inherited or *de novo*, and both types have been published as presenting similar frequencies (around 30-35%) (21). However, in the present series, the percentage of *de novo* variants were slightly higher (43.2%).

Evaluating their frequency by inheritance pattern and mutation type, we observed that, in contrast to other series (21, 28–30), inheritance pattern affected the prevalence or distribution of the mutation types, even not significantly. As expected, most of the pathogenic variants were found in the maternal allele, both in *de novo* and inherited cases. This is to be expected given that the clinic is much more overt in these patients and, therefore, our series is more abundant in patients with PHP1A/iPPSD2mat than in patients with PPHP/iPPSD2pat. A higher percentage of maternally inherited missense variants was observed, compared to the typically more frequent variants that generate truncated proteins observed in the case of *de novo* variants or variants of unknown origin. Interestingly, these percentages are reversed when, after determining the carrier allele in cases of *de novo* variants or variants of unknown origin, we re-evaluate the effect of the allele involved and the type of variant. In these cases, missense variants are especially frequent in the paternal allele. Also very striking is the high percentage of variants that we detect in the paternal allele in *de novo* cases (34.4%) or variants of unknown origin (23.1%) compared to the few cases that inherit the variant from their father (2%). These results are in concordance with previous papers referring that the risk of *de novo* mutation increases with paternal age (31, 32) due to their mitotic origin and the fact that additional mitoses are experienced by germ cells as fathers age increases (33).

Confirmation of which allele carries the alteration is, as we have mentioned, essential for the correct management of the patient. In our series we have confirmed that the clinical manifestations presented by the patients are concordant with those described for patients with the variant in their maternal or paternal allele (2) regardless of whether it was inherited or *de novo*. That is, patients with iPPSD2mat presented PTH resistance and manifestations of AHO regardless of whether the alteration had been inherited or not. However, in patients with variants in their paternal allele (iPPSD2pat), such resistance was absent.

Geneticists usually use the value of <1% to estimate the risk of recurrence for *de novo* mutations (34). However, with the implementation of NGS it has become clear that there is a higher than previously recognized contribution of “pre‐germline” mutational events in parents who themselves are mosaic and usually clinically unaffected (32, 33). Failure to identify low levels of mosaic mutations may lead to the misinterpretation of genetic testing results and to an incorrect genetic counselling. Specifically, a patient can be genetically underdiagnosed or their causative molecular alterations underestimated (35). Regarding familial implications inherited cases may be misinterpreted as sporadic due to low-level mosaicism in the carrier parents (36, 37), resulting in incorrect recurrence-risk assessment (33). So, bearing in mind the high percentage of *de novo* and unknown origin variants identified in our series and knowing that mosaicism for point mutations (36–38), long deletion (39) and chromosomal mosaicism had already been described (40) in iPPSD associated with *GNAS* region (even employing classical genetic analysis techniques) we considered essential the use of the NGS. Indeed, taking into account our previous experience, NGS enabled the detection of mosaicism in one of the progenitors of a patient who had been diagnosed as carrier of a variant of *de novo* origin (26).

As mentioned NGS technology allows for very high-fold coverage of sequenced fragments and the detection of low level mosaic mutations often classified as background noise and missed in Sanger sequencing (24). We use deep targeted NGS (average number of reads >500X) (41, 42) to enhance sensitivity and accuracy of the detection of known variants at the *GNAS* gene. In addition, we selected the potential carrier parent based on the identification of the allele presenting the alteration in the proband. Following this approach, we detected 8.11% germline mosaicism among the parents analyzed in our series, being two mothers and one father. One of the mothers had been previously described and, with 10% mosaicism, presented clinical features compatible with POH (26). The father with the same percentage of mosaicism, however, had no manifestations. In the case of the second mother, her percentage of mosaicism was much lower (2% in lymphocytes and about 1% in buccal mucosal cells), and she was also asymptomatic. In these cases of such low percentages of mosaicism, we considered essential to analyze independent tissues to validate the findings due to the high rate of sequencing errors in NGS, that cannot be discarded when searching for mosaicism.

The main limitation of this retrospective study is that only peripheral DNA was available for most of the parent patients tested. The availability of tissues of different embryonic origin (blood, saliva, dermal fibroblasts, and oral mucosa) would have helped to identify the presence of mosaicism in other tissue when not detected at lymphocytes’. Therefore, and although the series analyzed is relatively small, it is possible that the parental mosaicism associated with *GNAS* alterations is even greater than the identified 8.1%. We believe it would be important to replicate these studies in other series in order to estimate this percentage as it could provide precisions for transmission risk to offspring in genetic counseling. Besides, the availability of different tissues may also help to establish a correlation between the extent of the mosaic and the severity of the phenotype (if exists). Likewise, we should not lose sight of the fact that these results open the possibility that *GNAS* mosaicism may be the cause of iPPSD2 in patients in whom the mutation has not been identified by Sanger sequencing.

So, in conclusion, when an apparently *de novo* mutation is identified in a family, genetic mosaicism cannot be discarded as an underlying mechanism of the disease. The use of more sensitive techniques as NGS is essential for accurate recurrence-risk estimates as it allows the detection (or discard the presence) of low-level mosaic mutations. The identification of mosaic mutations in the proband or their parents is important in recurrence- and transmission-risk assessment. In addition to genetic counseling, the correct detection of a mosaic mutation is essential for disease diagnosis and management in iPPSD2 patients.

## Data availability statement

The data presented in the study are deposited in the European Nucleotide Archive (ENA) repository (https://www.ebi.ac.uk/ena), accession number PRJEB56694.

## Ethics statement

The studies involving human participants were reviewed and approved by Ethics committee for clinical research of Euskadi-Basque Country (CEIC-E). Written informed consent to participate in this study was provided by the participants’ legal guardian/next of kin.

## Author contributions

YV, AP, and GPN conceived and designed the study. Members of the Spanish Network for Imprinting Disorders provided blood samples, data acquisition, and clinical details of the patients. YV, AP, and AM-A, performed the genetic analysis. YV, AP, AM-A, and GPN analyzed and interpreted the data. YV, AP, and GPN wrote the manuscript. All authors reviewed and criticized it, approved the final version as submitted, and agreed to be accountable for all.

## References

[1] Thompson MD Hendy GN Percy ME Bichet DG Cole DEC . G Protein-coupled receptor mutations and human genetic disease. Methods Mol Biol (2014) 1175:153–87. doi: 10.1007/978-1-4939-0956-8_8 25150870

[2] Mantovani G Bastepe M Monk D de Sanctis L Thiele S Usardi A . Diagnosis and management of pseudohypoparathyroidism and related disorders: first international consensus statement. Nat Rev Endocrinol (2018) 14:476–500. doi: 10.1038/s41574-018-0042-0 29959430 PMC6541219

[3] Elli FM Pereda A Linglart A Perez de Nanclares G Mantovani G . Parathyroid hormone resistance syndromes - inactivating PTH/PTHrP signaling disorders (iPPSDs). Best Pract Res Clin Endocrinol Metab (2018) 32:941–54. doi: 10.1016/j.beem.2018.09.008 30665554

[4] Thiele S Mantovani G Barlier A Boldrin V Bordogna P De Sanctis L . From pseudohypoparathyroidism to inactivating PTH/PTHrP signalling disorder (iPPSD), a novel classification proposed by the EuroPHP network. Eur J Endocrinol (2016) 175:P1–P17. doi: 10.1530/EJE-16-0107 27401862

[5] Albright F Burnett CH Smith PH Parson W . Pseudohypoparathyroidism - an example of “Seabright-bantam syndrome” Endocrinology (1942) 30:922–32. doi: 10.1016/S0022-3476(50)80173-7

[6] Albright F Forbes AP Henneman PH . Pseudo-pseudohypoparathyroidism. TransAssoc Am Physicians (1952) 65:337–50. 13005676

[7] Pignolo RJ Ramaswamy G Fong JT Shore EM Kaplan FS . Progressive osseous heteroplasia: diagnosis, treatment, and prognosis. Appl Clin Genet (2015) 8:37–48. doi: 10.2147/TACG.S51064 25674011 PMC4321643

[8] Turan S Thiele S Tafaj O Brix B Atay Z Abali S . Evidence of hormone resistance in a pseudo-pseudohypoparathyroidism patient with a novel paternal mutation in. GNAS. Bone (2015) 71:53–7. doi: 10.1016/j.bone.2014.10.006 PMC4273232 25464124

[9] Swaroop A Agarwal N Gruen JR Bick D Weissman SM . Differential expression of novel gsα signal transduction protein cDNA species. Nucleic Acids Res (1991) 19:4725–9. doi: 10.1093/nar/19.17.4725 PMC328715 1716359

[10] Ischia R Lovisetti-Scamihorn P Hogue-Angeletti R Wolkersdorfer M Winkler H Fischer-Colbrie R . Molecular cloning and characterization of NESP55, a novel chromogranin-like precursor of a peptide with 5-HT1B receptor antagonist activity. J Biol Chem (1997) 272:11657–62. doi: 10.1074/jbc.272.17.11657 9111083

[11] Kehlenbach RH Matthey J Huttner WB . XL Alpha s is a new type of G protein. Nature (1994) 372:804–9. doi: 10.1038/372804a0 7997272

[12] Bastepe M . The *GNAS* locus: Quintessential complex gene encoding gsalpha, XLalphas, and other imprinted transcripts. Curr Genomics (2007) 8:398–414. doi: 10.2174/138920207783406488 19412439 PMC2671723

[13] Hayward BE Moran V Strain L Bonthron DT . Bidirectional imprinting of a single gene: *GNAS*1 encodes maternally, paternally, and biallelically derived proteins. Proc Natl Acad Sci U.S.A. (1998) 95:15475–80. doi: 10.1073/pnas.95.26.15475 PMC28067 9860993

[14] Turan S Bastepe M . *GNAS* spectrum of disorders. Curr Osteopor Rep (2015) 13:146–58. doi: 10.1007/s11914-015-0268-x PMC4417430 25851935

[15] Hayward BE Kamiya M Strain L Moran V Campbell R Hayashizaki Y . The human *GNAS*1 gene is imprinted and encodes distinct paternally and biallelically expressed G proteins. Proc Natl Acad Sci U.S.A. (1998) 95:10038–43. doi: 10.1073/pnas.95.17.10038 PMC21457 9707596

[16] Hayward BE Bonthron DT . An imprinted antisense transcript at the human *GNAS*1 locus. Hum Mol Genet (2000) 9:835–41. doi: 10.1093/hmg/9.5.835 10749992

[17] Mantovani G Ballare E Giammona E Beck-Peccoz P Spada A . The gsα gene: Predominant maternal origin of transcription in human thyroid gland and gonads. J Clin Endocrinol Metab (2002) 87:4736–40. doi: 10.1210/jc.2002-020183 12364467

[18] Yu S Yu D Lee E Eckhaus M Lee R Corria Z . Variable and tissue-specific hormone resistance in heterotrimeric gs protein alpha-subunit (Gsalpha) knockout mice is due to tissue-specific imprinting of the gsalpha gene. Proc Natl Acad Sci U.S.A. (1998) 95:8715–20. doi: 10.1073/pnas.95.15.8715 PMC21142 9671744

[19] Tafaj O Hann S Ayturk U Warman ML Jüppner H . Mice maintain predominantly maternal gαs expression throughout life in brown fat tissue (BAT), but not other tissues. Bone (2017) 103:177–87. doi: 10.1016/j.bone.2017.07.001 PMC5943706 28694163

[20] Hayward BE Barlier A Korbonits M Grossman AB Jacquet P Enjalbert A . Imprinting of the g(s)alpha gene *GNAS*1 in the pathogenesis of acromegaly. J Clin Invest (2001) 107:R31–6. doi: 10.1172/JCI11887 PMC208949 11254676

[21] Elli FM Linglart A Garin I de Sanctis L Bordogna P Grybek V . The prevalence of *GNAS* deficiency-related diseases in a Large cohort of patients characterized by the EuroPHP network. J Clin Endocrinol Metab (2016) 101:3657–68. doi: 10.1210/jc.2015-4310 27428667

[22] Mantovani G Linglart A Garin I Silve C Elli FM de Nanclares GP . Clinical utility gene card for: pseudohypoparathyroidism. Eur J Hum Genet (2013) 21. doi: 10.1038/ejhg.2012.211 PMC3658187 22968134

[23] Moog U Felbor U Has C Zirn B . Disorders caused by genetic mosaicism. Dtsch Arztebl Int (2020) 116:119–25. doi: 10.3238/arztebl.2020.0119 PMC7081367 32181732

[24] Rohlin A Wernersson J Engwall Y Wiklund L Björk J Nordling M . Parallel sequencing used in detection of mosaic mutations: Comparison with four diagnostic DNA screening techniques. Hum Mutat (2009) 30:1012–20. doi: 10.1002/humu.20980 19347965

[25] Qin L Wang J Tian X Yu H Truong C Mitchell JJ . Detection and quantification of mosaic mutations in disease genes by next-generation sequencing. J Mol Diagn (2016) 18:446–53. doi: 10.1016/j.jmoldx.2016.01.002 26944031

[26] Pereda A Martos-Tello JM Garin I Errea-Dorronsoro J Perez de Nanclares G . Progressive osseous heteroplasia caused by a mosaic GNAS mutation. Clin Endocrinol (Oxf) (2018) 88:993–5. doi: 10.1111/cen.13584 29464731

[27] Robinson JT Thorvaldsdóttir H Winckler W Guttman M Lander ES Getz G . Integrative genomics viewer. Nat Biotechnol (2011) 29:24–6. doi: 10.1038/nbt.1754 PMC3346182 21221095

[28] Elli FM deSanctis L Ceoloni B Barbieri AM Bordogna P Beck-Peccoz P . Pseudohypoparathyroidism type ia and pseudo-pseudohypoparathyroidism: the growing spectrum of *GNAS* inactivating mutations. Hum Mutat (2013) 34:411–6. doi: 10.1002/humu.22265 23281139

[29] Thiele S Werner R Grötzinger J Brix B Staedt P Struve D . A positive genotype–phenotype correlation in a large cohort of patients with pseudohypoparathyroidism type ia and pseudo-pseudohypoparathyroidism and 33 newly identified mutations in the *GNAS* gene. Mol Genet Genom Med (2015) 3:111–20. doi: 10.1002/mgg3.117 PMC4367083 25802881

[30] Lemos MC Thakker RV . *GNAS* mutations in pseudohypoparathyroidism type 1a and related disorders. Hum Mutat (2015) 36:11–9. doi: 10.1002/humu.22696 PMC4309471 25219572

[31] Kong A Frigge ML Masson G Besenbacher S Sulem P Magnusson G . Rate of *de novo* mutations and the importance of father’s age to disease risk. Nature (2012) 488:471–5. doi: 10.1038/nature11396 PMC3548427 22914163

[32] Zemet R Van den Veyver IB Stankiewicz P . Parental mosaicism for apparent *de novo* genetic variants: Scope, detection, and counseling challenges. Prenat Diagn (2022) 42:811–21. doi: 10.1002/pd.6144 PMC9995893 35394072

[33] Campbell IM Stewart JR James RA Lupski JR Stankiewicz P Olofsson P . Parent of origin, mosaicism, and recurrence risk: Probabilistic modeling explains the broken symmetry of transmission genetics. Am J Hum Genet (2014) 95:345–59. doi: 10.1016/j.ajhg.2014.08.010 PMC4185125 25242496

[34] Röthlisberger B Kotzot D . Recurrence risk in de novo structural chromosomal rearrangements. Am J Med Genet A (2007) 143A:1708–14. doi: 10.1002/ajmg.a.31826 17603796

[35] Elli FM de Sanctis L Bergallo M Maffini MA Pirelli A Galliano I . Improved molecular diagnosis of McCune-albright syndrome and bone fibrous dysplasia by digital PCR. Front Genet (2019) 10:862. doi: 10.3389/fgene.2019.00862 31620168 PMC6760069

[36] Wang Q Xian J Chen P Wang J Gao Y Zheng X . A novel *GNAS* mutation inherited from probable maternal mosaicism causes two siblings with pseudohypoparathyroidism type 1A. J Pediatr Endocrinol Metab (2020) 33:1219–24. doi: 10.1515/jpem-2019-0476 32866120

[37] Ngai YF Chijiwa C Mercimek-Mahmutoglu S Stewart L Yong S-LL Robinson WP . Pseudohypoparathyroidism type 1a and the *GNAS* p.R231H mutation: Somatic mosaicism in a mother with two affected sons. Am J Med Genet A (2010) 152A:2784–90. doi: 10.1002/ajmg.a.33172 20979189

[38] Aldred MA Bagshaw RJ Macdermot K Casson D Murch SH Walker-Smith JA . Germline mosaicism for a *GNAS*1 mutation and albright hereditary osteodystrophy. J Med Genet (2000) 37:E35. doi: 10.1136/jmg.37.11.e35 11073544 PMC1734481

[39] Fernandez-Rebollo E García-Cuartero B Garin I Largo C Martínez F Garcia-Lacalle C . Intragenic *GNAS* deletion involving exon A/B in pseudohypoparathyroidism type 1A resulting in an apparent loss of exon A/B methylation: potential for misdiagnosis of pseudohypoparathyroidism type 1B. J Clin Endocrinol Metab (2010) 95:765–71. doi: 10.1210/jc.2009-1581 PMC2840867 20008020

[40] Maupetit-Mehouas S Mariot V Reynes C Bertrand G Feillet F Carel J-C . Quantification of the methylation at the *GNAS* locus identifies subtypes of sporadic pseudohypoparathyroidism type ib. J Med Genet (2011) 48:55–63. doi: 10.1136/jmg.2010.081356 20972248

[41] Dai C Cheng D Li W Zeng S Lu G Zhang Q . Identification of paternal germline mosaicism by MicroSeq and targeted next-generation sequencing. Mol Genet Genom Med (2020) 8:e1394. doi: 10.1002/mgg3.1394 PMC7507370 32643877

[42] Grotta S D’Elia G Scavelli R Genovese S Surace C Sirleto P . Advantages of a next generation sequencing targeted approach for the molecular diagnosis of retinoblastoma. BMC Cancer (2015) 15:841. doi: 10.1186/s12885-015-1854-0 26530098 PMC4632486

