# Supplementary material for: Frequency of de novo variants and parental mosaicism in families with inactivating PTH/PTHrP signaling disorder type 2
Source: Front Endocrinol (Lausanne). 2023 Jan 4;13:1055431. doi: 10.3389/fendo.2022.1055431 (PMC9846528; doi:10.3389/fendo.2022.1055431)
Supplement: Supplementary file 1 [file Table_1.docx]

Supplementary Material

# Supplementary Tables

**Supplementary Table 1.** Clinical and molecular characteristics of the patients of the complete cohort. The clinical information collects the presence or absence of the major and minor criteria of the iPPSD classification. Non-available refers to those index patients for whom no sample was available for present studies. Unresolved indicates that, despite having performed the different experiments described in the M&M section, we were unable to identify the carrier allele.* Not paternal, maternal inferred; # Positive control; ‡ Variant at exon 1.

| Patient code | Type of variant | Inheritance | Involved allele | Sex | Age at diagnosis | PTHr | Ectopic ossification | Brachydactyly | TSH resistance | Other hormonal resistance | Motor and cognitive retardation or impairment | IUGR and postnatal growth retardation | Obesity / overweight | Flat nasal brigde and/or maxillar hypoplasia and/or round face |
| --- | --- | --- | --- | --- | --- | --- | --- | --- | --- | --- | --- | --- | --- | --- |
| PHP0001 | Frameshift | De novo | Non-available | F | 1.5 | Yes | Yes | Yes | Yes | Yes | No data | Yes | No | Yes |
| PHP0004 | Missense | Maternal |  | M | 3.6 | Yes | No data | Yes | Yes | No | No data | No | Yes | Yes |
| PHP0006 | Gross deletion | Maternal |  | F | 3.3 | Yes | Yes | Yes | Yes | No | No data | No | No | Yes |
| PHP0007 | Frameshift | Maternal |  | M | 2.2 | Yes | Yes | Yes | Yes | No | No data | No | Yes | Yes |
| PHP0008 | Nonsense | Maternal |  | M | 1.6 | Yes | No data | Yes | Yes | No data | No data | Yes | No | Yes |
| PHP0010 | Nonsense | Unknown | Non-available | F | 27.7 | Yes | Yes | Yes | Yes | No data | No data | Yes | Yes | Yes |
| PHP0012 | Nonsense | Maternal |  | M | 2 | Yes | Yes | Yes | Yes | No | No data | No | No | Yes |
| PHP0016 | Nonsense | De novo | Non-available | F | 1.1 | Yes | Yes | Yes | Yes | No data | No data | Yes | Yes | Yes |
| PHP0023 | Missense | Maternal |  | M | 14.25 | Yes | No | Yes | Yes | No data | No data | No | Yes | Yes |
| PHP0024 | Nonsense | De novo | Non-available | F | 7.8 | Yes | Yes | Yes | Yes | No data | No data | No | Yes | Yes |
| PHP0026 | Missense | Maternal |  | F | 18.2 | Yes | No | Yes | Yes | Yes | No data | No | Yes | Yes |
| PHP0027 | Missense | De novo | Non-available | M | 13.6 | Yes | No | Yes | Yes | No | No data | Yes | No | Yes |
| PHP0033 | Splicing | Maternal |  | M | 14.2 | Yes | No | Yes | Yes | No | No data | Yes | No data | No data |
| PHP0040 | Frameshift | Unknown | Non-available | M | 12 | Yes | No | No | Yes | No data | No data | Yes | No data | No data |
| PHP0050 | Missense | De novo | Non-available | F | 11.4 | Yes | No data | Yes | No data | No data | No data | No | No data | Yes |
| PHP0061 | Inversion | De novo | Non-available | M | 5.4 | Yes | No | Yes | Yes | No | No data | Yes | Yes | No |
| PHP0064 | Splicing | Maternal |  | M | 11 | Yes | No | Yes | Yes | No data | No data | No | Yes | Yes |
| PHP0067 | Frameshift | Maternal |  | M | 1 | Yes | Yes | Yes | Yes | No data | No data | No | Yes | Yes |
| PHP0073 | Nonsense | Unknown | Non-available | F | 20 | Yes | Yes | Yes | Yes | No | No data | No | Yes | No |
| PHP0075 | In-frame duplication | De novo | Non-available | M | 14 | Yes | No data | Yes | Yes | No data | No data | No data | No | No |
| PHP0076 | Frameshift | De novo | Non-available | M | 7.9 | Yes | Yes | Yes | Yes | No data | No data | No | Yes | No data |
| PHP0077 | Frameshift | Maternal |  | M | 3.1 | Yes | Yes | Yes | Yes | No data | No data | No | Yes | Yes |
| PHP0083 | Frameshift | Unknown | Non-available | F | 12.3 | Yes | Yes | Yes | Yes | No data | No data | No | No data | No data |
| PHP0085 | Frameshift | De novo | Maternal* | F | 3.1 | Yes | Yes | Yes | Yes | No data | No data | No data | Yes | Yes |
| PHP0086 | Missense | Maternal |  | F | 24 | Yes | No data | Yes | Yes | No data | No data | No | No data | No data |
| PHP0092 | Frameshift | De novo | Non-available | M | 10 | Yes | Yes | Yes | Yes | Yes | Yes | Yes | Yes | Yes |
| PHP0093 | Missense | Unknown | Non-available | M | 16 | No data | No data | No data | No data | No data | No data | No data | No data | No data |
| PHP0094 | Frameshift | Unknown | Non-available | F |  | No data | No data | No data | No data | No data | No data | No data | No data | No data |
| PHP0108 | Frameshift | Unknown | Non-available | F | 6.5 | Yes | Yes | No data | Yes | No | No data | No data | No data | No data |
| PHP0109 | Frameshift | Maternal |  | F | 1.5 | Yes | Yes | No data | Yes | No | Yes | No data | Yes | No data |
| PHP0111 | Missense | Maternal |  | M | 2.7 | Yes | No data | No data | Yes | No data | Yes | Yes | Yes | Yes |
| PHP0115 | Missense | Unknown | Maternal* | F | 5 | Yes | No data | Yes | Yes | Yes | Yes | Yes | Yes | Yes |
| PHP1003 | Frameshift | Unknown | Maternal* | M | 25 | Yes | Yes | No | Yes | Yes | No | No | No | No |
| PHP1006 | Nonsense | Unknown | Non-available | M |  | No data | No data | No data | No data | No data | No data | No data | No data | No data |
| PHP1015 | Nonsense | De novo | Unsolved | M | 11 | Yes | Yes | Yes | Yes | No | Yes | Yes | No | Yes |
| PHP1016 | Gross deletion | Maternal |  | M | 2 | Yes | Yes | Yes | Yes | Yes | Yes | Yes | Yes | Yes |
| PHP1021 | Frameshift | De novo | Maternal* | M | 9 | Yes | Yes | Yes | Yes | No | No data | Yes | No data | No data |
| PHP1022 | Frameshift | De novo | Unsolved | F | 16 | Yes | Yes | No data | Yes | No | No data | No | Yes | Yes |
| PHP1025 | Missense | De novo | Paternal | M | 6.5 | No | No data | Yes | Yes | No | Yes | Yes | Yes | Yes |
| PHP1046 | Splicing | De novo | Unsolved | F | 4 | Yes | Yes | Yes | Yes | No | No data | No | Yes | Yes |
| PHP1047 | Missense | De novo | Maternal* | F | 2 | Yes | Yes | Yes | Yes | No | Yes | Yes | Yes | Yes |
| PHP1049 | Missense | De novo | Paternal | F | 5 | No | No | Yes | No | No | No | Yes | No | No data |
| PHP1051 | Splicing | De novo | Unsolved | F | 1 | Yes | No data | Yes | Yes | No | No | Yes | No | Yes |
| PHP1061 | Missense | Unknown | Non-available | M |  | No data | No data | No data | No data | No data | No data | No data | No data | No data |
| PHP1065 | Missense | Maternal |  | M | 4 | Yes | No data | Yes | Yes | No data | Yes | Yes | Yes | Yes |
| PHP1078 | Splicing | De novo | Maternal* | F | 30 | Yes | Yes | No data | Yes | Yes | No data | No data | No data | No data |
| PHP1088 | Missense | Maternal |  | M | 13 | Yes | No data | Yes | Yes | No | Yes | Yes | Yes | Yes |
| PHP1089 | Frameshift | De novo | Paternal | M | 6.25 | No | Yes | Yes | No | No | No | Yes | No | No |
| PHP1095 | Nonsense | Unknown | Unsolved‡ | F | 9 | No | Yes | Yes | No | No | No data | Yes | Yes | No |
| PHP1097 | Missense | Unknown | Maternal* | F | 3.5 | Yes | No data | Yes | Yes | No | No data | No | Yes | Yes |
| PHP1106 | Missense | Maternal |  | M | 5 | Yes | No data | Yes | Yes | No | Yes | Yes | Yes | Yes |
| PHP1108 | Frameshift | De novo | Unsolved | F | 5 | Yes | Yes | Yes | No data | No data | Yes | Yes | Yes | Yes |
| PHP1114 | Frameshift | De novo | Maternal* | F | 9 | Yes | Yes | Yes | Yes | No | Yes | Yes | Yes | Yes |
| PHP1120 | Nonsense | Maternal |  | F |  | No data | No data | No data | No data | No data | No data | No data | No data | No data |
| PHP1122 | Missense | Maternal |  | F | 28 | Yes | No data | Yes | Yes | Yes | Yes | Yes | Yes | Yes |
| PHP1124 | Frameshift | Unknown | Maternal* | F | 13 | Yes | No | Yes | No | Yes | No | Yes | No data | No data |
| PHP1125 | Splicing | De novo | Maternal# | M | 3 | Yes | Yes | Yes | Yes | No | Yes | Yes | Yes | Yes |
| PHP1126 | Frameshift | De novo | Maternal* | F | 12 | Yes | Yes | Yes | Yes | No | No data | Yes | Yes | Yes |
| PHP1129 | Frameshift | Unknown | Maternal* | F | 9 | Yes | No data | Yes | Yes | No data | Yes | No data | No data | Yes |
| PHP1151 | Frameshift | De novo | Maternal* | M | 2 | Yes | Yes | Yes | Yes | No | Yes | Yes | Yes | Yes |
| PHP1153 | Splicing | Maternal |  | F | 5 | Yes | Yes | Yes | Yes | No data | Yes | No data | Yes | Yes |
| PHP1155 | Nonsense | De novo | Unsolved‡ | F | 11 | No | Yes | No | No | No | No | Yes | No | No |
| PHP1156 | Missense | Unknown | Unsolved | M | 6 | Yes | No | Yes | Yes | Yes | Yes | No data | Yes | Yes |
| PHP1166 | Frameshift | Maternal |  | M | 2.4 | Yes | No | No data | Yes | No | No | No | Yes | Yes |
| PHP1170 | Missense | De novo | Paternal | M | 10 | No | Yes | Yes | No data | No | Yes | Yes | Yes | Yes |
| PHP1175 | Frameshift | Maternal |  | F | 3.5 | Yes | Yes | Yes | Yes | No | Yes | Yes | No | Yes |
| PHP1179 | Nonsense | De novo | Paternal | M | 11 | No | Yes | Yes | No | No | No data | No | Yes | Yes |
| PHP1180 | In-frame duplication | Paternal |  | F | 11.3 | No | No | Yes | Yes | No | No | Yes | No | No data |
| PHP1183 | Missense | Unknown | Paternal | F | 18 | No | No data | Yes | No | No | Yes | Yes | No | Yes |
| PHP1188 | Missense | Unknown | Paternal | F | 10.5 | No | No data | Yes | No | No | No data | Yes | No | No data |
| PHP1190 | Missense | De novo | Maternal* | F | 1 | Yes | No data | No data | Yes | No data | Yes | Yes | Yes | No data |
| PHP1191 | Missense | De novo | Paternal | M | 30 | No | No data | Yes | Yes | No | Yes | Yes | No data | Yes |
| PHP1193 | Missense | Unknown | Non-available | F |  | Yes | No data | Yes | Yes | No data | No data | Yes | Yes | No data |
| PHP1196 | Splicing | De novo | Paternal | M | 17 | No | No data | Yes | No data | No data | Yes | Yes | No data | No data |
| PHP1203 | Splicing | Unknown | Maternal* | M | 30 | Yes | No data | Yes | Yes | No data | Yes | No data | No data | No data |
| PHP1204 | Gross deletion | Unknown | Paternal | F | 11 | No | Yes | No data | No data | No data | No data | Yes | Yes | No data |
| PHP1206 | Frameshift | De novo | Maternal* | F |  | No data | No data | No data | No data | No data | No data | No data | No data | No data |
| PHP1215 | Missense | Maternal |  | M | 6.3 | Yes | No data | No data | Yes | No data | Yes | Yes | No data | No data |
| PHP1217 | Frameshift | De novo | Paternal | F | 2.3 | No | Yes | No | No | No | No data | No data | No | No |
| PHP1220 | Gross deletion | De novo | Maternal | M | 3.4 | Yes | Yes | No data | No data | No data | No data | No data | Yes | Yes |
| PHP1223 | Splicing | Unknown | Unsolved‡ | F | 32 | Yes | No data | Yes | Yes | Yes | No | Yes | Yes | No data |
| PHP1237 | Missense | De novo | Paternal | F | 12 | No | Yes | Yes | Yes | No | No | Yes | No | No |
| PHP1242 | Frameshift | Maternal |  | F | 2.1 | Yes | Yes | Yes | Yes | No data | Yes | Yes | Yes | Yes |
| PHP1244 | Frameshift | De novo | Maternal* | F | 1.7 | Yes | Yes | No | Yes | No | Yes | No | Yes | Yes |
| PHP1249 | Missense | De novo | Maternal | F | 2.5 | Yes | No data | Yes | Yes | No | Yes | No | Yes | No data |
| PHP1251 | Gross deletion | Paternal |  | F | 2.75 | No | No | Yes | Yes | No data | Yes | Yes | No | No data |
| PHP1267 | Missense | Maternal |  | F | 3 | Yes | No | Yes | Yes | No | Yes | No | Yes | Yes |
| PHP1283 | Missense | Maternal |  | F | 1.8 | Yes | No data | No | No | No data | No data | No data | Yes | No data |
| PHP1288 | Frameshift | Unknown | Non-available | M | 11.2 | Yes | Yes | Yes | Yes | Yes | No | Yes | Yes | No |
| PHP1289 | Nonsense | De novo | Unsolved‡ | F | 6 | Yes | Yes | No | Yes | No data | Yes | No | Yes | No data |
| PHP1299 | Nonsense | Unknown | Maternal* | M | 11.8 | Yes | Yes | Yes | Yes | No data | No data | Yes | No data | No data |
| PHP1314 | In-frame duplication | De novo | Paternal | F | 13 | No data | No data | Yes | Yes | No data | No | Yes | No data | No data |
| PHP1319 | Missense | De novo | Paternal | M | 2 | No | Yes | Yes | No | No | No | Yes | No | No |
| PHP1320 | Frameshift | De novo | Maternal* | F | 12 | Yes | Yes | Yes | No | No | Yes | No | Yes | Yes |
| PHP1322 | Frameshift | Maternal |  | M | 11.2 | Yes | No | Yes | Yes | No data | Yes | Yes | Yes | Yes |

**Supplementary Table 2**. The sequence of the primers used for the study of the GNAS locus by RT-PCR. Primers FAB_cDNA + R13_cDNA were used to amplify the paternal allele including exon 2 to 13 of GNAS gene, whereas FNESP55_cDNA + R13_cDNA allowed the amplification of the maternal one. Combinations of primers F2_cDNA to R8_cDNA were used, when needed, for nested PCRs. F: forward, R: reverse.

| NAME | SEQUENCE (5’🡪3’) |
| --- | --- |
| FAB_cDNA | GCTGGAGCGAGCCCCTGT |
| FNESP55_cDNA | GAAGGAGCCCAAGGAGGAGAAGCAGCGGC |
| R13_cDNA | GGTGAAATGAGGGTAGCAGT |
|  |  |
| F2_cDNA | GATGAGGATCCTGCATGTT |
| F5_cDNA | CCTGACTTTGACTTCCCT |
| R6_cDNA | CCTTGGCATGCTCATAGAATTC |
| F6_cDNA | GCTCCAACGAGTACCAGCT |
| R7_cDNA | GCTCGGCACATAGTCAGCCT |
| R8_cDNA | GGAAGTTGACTTTGTCCA |

**Supplementary Table 3**. Primers used for ASO-RT-PCR of GNAS gene for variants located at GNAS exon 1. The FE1_rs7121 primer is located at the 5’ end of exon 1 of GNAS, whereas both RE1 primers anneal with one of the different variants of the SNP (C or T).

| NAME | SEQUENCE (5’🡪3’) |
| --- | --- |
| FE1_rs7121 | CTGCCTCGGGAACAGTAAG |
| RE1_rs7121C | GCACGTTCATCACACTCATG |
| RE1_rs7121T | GCACGTTCATCACACTCATA |

## Supplementary Figure


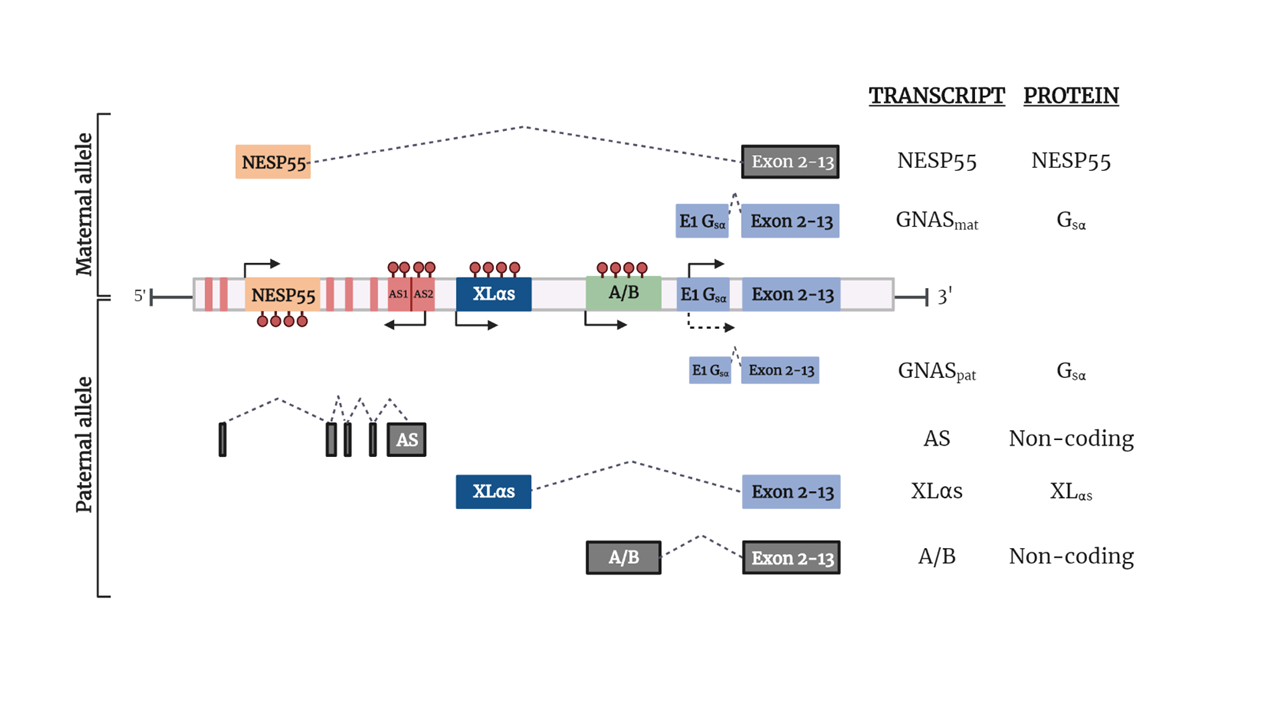


**Supplementary Figure.** **Schematic representation of the GNAS locus and its multiple transcripts (20q13.3). Gsα, XLαs, and NESP55 are the main coding transcripts of the GNAS locus**. In addition, AS and A/B are non-coding transcripts. These distinct transcripts are generated through alternative first exons that splice onto common 2-13 exons of GNAS gene. The first exons are regulated by imprinting in differentially methylated regions (DMRs). The red lollipops represent the methylation of the DMRs, so these transcripts’ expression is restricted to maternal (above) or paternal (below) allele, except of Gsα which is not regulated by genomic imprinting and its expression is biallelic in most tissues, even if in some of them it is predominantly maternal. Dashed lines indicate alternative splicing; colored boxes, exons; and gray colored boxes, non-coding regions. The arrows indicate the orientation of the transcription.
